# Decreased Expression of MiRNA-204-5p Contributes to Glioma Progression and Promotes Glioma Cell Growth, Migration and Invasion

**DOI:** 10.1371/journal.pone.0132399

**Published:** 2015-07-02

**Authors:** Zhiqiang Xia, Fang Liu, Jian Zhang, Li Liu

**Affiliations:** 1 Department of Microbiology, Institute of Basic Medical Sciences, Chinese Academy of Medical Sciences, Beijing 100730, China; 2 School of Basic Medicine, Peking Union Medical College, Beijing 100730, China; 3 Suzhou Wuzhong Hospital, Suzhou 210031, China; University of Kentucky College of Medicine, UNITED STATES

## Abstract

Gliomas are the most common malignant primary brain tumors in adults and exhibit a spectrum of aberrantly aggressive phenotype. Although increasing evidence indicated that the deregulation of microRNAs (miRNAs) contributes to tumorigenesis and invasion, little is known about the roles of miR-204-5p in human gliomas. In the present study, the expression of miR-204-5p in clinical glioma tissues was measured by qRT-PCR. The effects of miR-204-5p on glioma cell growth and metastasis were examined by overexpressing or inhibiting miR-204-5p. We found that the expression level of miR-204-5p was significantly reduced in clinical glioma tissues compared with normal brain tissues. Moreover, we revealed that the introduction of miR-204-5p dramatically suppressed glioma cell growth, migration and invasion. Furthermore, mechanistic investigations revealed that RAB22A, a member of the RAS oncogene family, is a direct functional target of miR-204-5p in gliomas. *In vivo*, restoring miR-204-5p expression in glioma cells suppressed tumorigenesis and increased overall host survival. Our findings suggest that miR-204-5p is a cancer suppressor miRNA and overexpression of miR-204-5p is a novel glioma treatment strategy.

## Introduction

High-grade gliomas are the most common form of adult malignant brain tumors, and are characterized by rapid progression, high resistance to radiotherapy or chemotherapy, as well as exhibit extremely poor clinical prognosis [1–4]. Despite recent advances in both diagnostic methods and therapeutic strategies including maximal safe surgical resection combined with standard radiation therapy and chemotherapy, many patients succumb to the disease within 5 years of diagnosis [5]. Consequently, a better understanding of the molecular mechanisms involving glioma formation and development will be helpful to highlight novel therapeutic targets and develop strategies for the treatment of gliomas.

Recent work has suggested that deregulation of a group of small non-coding RNAs called microRNAs (miRNAs) are involved in initiation and progression of many cancers [6, 7] including gliomas [8–10]. Several deregulated miRNAs target key gene products involved in cell proliferation, invasion, migration as well as chemo- and radio-resistance [11–14], which suggests that miRNAs are excellent biomarker candidates and potential therapeutic tools [15–17]. In gliomas, many miRNAs show aberrant expression patterns [18–20], and some have been shown to be involved in tumorigenesis by targeting tumor-associated genes [21–24]. In addition, miRNAs appear to be promising tumor biomarkers for glioma [25, 26]. Several studies have also shown that miR-204-5p is frequently downregulated in papillary thyroid carcinoma, gastric cancer, colorectal cancer and endometrial carcinoma, suggesting a common role of miR-204-5p in human tumorigenesis [27–30]. However, the roles of miR-204-5p in glioma remain undefined.

In the present study, we showed that downregulation of miR-204-5p in human gliomas is correlated with poor patient prognosis, and overexpression of miR-204-5p can inhibit the proliferation, migration and invasion of glioma cells *in vitro* and *in vivo*. Further mechanistic investigations revealed that miR-204-5p inhibits gliomas cell growth, migration and invasion by directly targeting RAB22A (a member of the RAS oncogene family), which appears to be a new prognostic factor in gliomas. Our data provide new insights into the molecular function of miR-204-5p in gliomas.

## Materials and Methods

### Cell culture

Two different PTEN background human malignant glioma cell lines LN-229 (with wild-type PTEN) and U87 (with PTEN loss) [31]were purchased from American Type Culture Collection (Rockville, MD), and cultured as recommended as monolayers in Dulbecco's modified Eagle's medium (Gibco, Los Angeles, CA, USA) supplemented with 10% fetal bovine serum (Gibco), in a humidified incubator at 37°C in a 5% CO_2_ atmosphere.

### Tissue samples

A total of 35 paraffin-embedded glioma and 10 normal brain samples were obtained from the Wuzhong Hospital, including 15 grade II tumors, 13 grade III tumors and 7 grade IV tumors according to WHO classification. These glioma cases were from 21 males and 14 females with age ranging from 17–79 years. The purpose and content of this study were explained fully to all subjects, whose signed informed consent was obtained prior to enrollment. Written informed consent was obtained from each patient or the next of kin, caretakers, or guardians on behalf of the minors, and the use of human tissue specimens was approved by the Institutional Review Board for Human Research at Wuzhong Hospital, China. All specimens had confirmed pathological diagnosis and were classified according to the WHO criteria. The protocol of this study was specifically approved by the Human Studies Committee of Wuzhong Hospital, Suzhou, China, in accordance with the guidelines of the Helsinki Declaration and the Human Studies Committee (approval number: 017).

### RNA isolation, reverse transcription and qRT–PCR

Total RNA was extracted from glioma tissues and normal brain tissues by Trizol Reagent (Invitrogen, Carlsbad, CA, USA). For miR-204-5p detection, reverse-transcribed complementary DNA was synthesized with the PrimeScript RT reagent Kit (TaKaRa, Dalian, China), and quantitative real time–PCR (qRT–PCR) was performed with SYBR Premix ExTaq (TaKaRa, Dalian, China) with the Stratagene Mx3000P real-time PCR system (Agilent Technologies, Inc., Santa Clara, CA, USA). Primer sequences of miR-204-5p are 5'GCCAGATCTGGAAGAAGATGGTGGTTAGT3' (forward) and 5'GGCGAATTCACAGTTGCCTACAGTATTCA3' (reverse). Expression levels were normalized against the endogenous snRNA U6 control. The relative expression ratio of miR-204-5p was calculated by the 2^−ΔΔCT^ method. For mRNA analyses, cDNA was synthesized using Moloney murine leukemia virus reverse transcriptase (Promega, Madison, WI, USA). qRT–PCR was performed with SYBR Premix ExTaq with the Stratagene Mx3000P real-time PCR system. β-actin was used as internal controls for mRNA quantification. The relative expression ratio of mRNA was calculated by the 2^−ΔΔCT^ method. PCR reactions for each gene were repeated three times. Independent experiments were done in triplicate.

### *In situ* hybridization

Using sense locked nucleic acid (LNA)-modified oligonucleotide probes, *in situ* hybridization was performed on paraffin-embedded sections (4μm thickness) of glioma specimens with an *in situ* hybridization kit (Boster Biological Technology, Ltd., Wuhan, China). After processing with 3% H_2_O_2_, sections were treated with proteinase K (2μg/ml) at 37°C for 30min, washed, and prehybridized for 2h at 37°C. Hybridization with digoxygenin (DIG)-labeled miRCURY LNA probes (probe sense: 5′-ACGCAGAGCCCGAAAGCCCCCAGT-3′) was performed overnight at 37 °C. Slides were then washed at 37 °C and incubated with alkaline phosphatase–conjugated sheep anti-DIG Fab fragments for 1h at room temperature. Staining was visualized by adding BM purple AP substrate (Roche, Basel, Switzerland) according to the manufacturer’s instructions.

### Establishment of glioma cell lines with stable expression of miR-204-5p

Lentiviral vectors which overexpress miR-204-5p were purchased from GeneChem (Shanghai, China). A lentiviral vector expressing scrambled RNA was used as the control and the sequence was 5′-TTCTCCGAACGTGTCACGT-3′. LN229 and U87 cells were infected with lentiviral vector, and polyclonal cells with green fluorescent protein signals were selected for further experiments using fluorescence-activated cell sorting flow cytometry. Total RNA from these cell clones was isolated, and levels of miR-204-5p were quantified using qRT–PCR.

### MTT assay

Cell viability was analyzed using MTT assay. Cells were seeded in 96-well plates at a density of 1000–1500 cells/well and incubated for 1, 2, 3, 4 or 5 days. Approximately 20μl of MTT (5mg/ml; Sigma, St Louis, MO, USA) was added to each well and incubated for 4h. At the end of incubation, supernatants were removed, and 150μl of dimethyl sulfoxide (Sigma) was added to each well. The absorbance value (optical density) of each well was measured at 490nm. For each experimental condition, 10 wells were used. All experiments were performed thrice.

### EdU proliferation assay

The proliferation of LN229 and U87 cells were examined using the Cell-Light EdU Apollo488 *In Vitro* Imaging Kit (RiboBio) according to the manufacturer’s protocol. Briefly, cells were incubated with 10μM EdU for 2h before fixation with 4% paraformaldehyde, permeabilization by 0.3% Triton X-100 and EdU staining. Cell nuclei were stained with 5μg/ml DAPI (4′,6-diamidino-2-phenylindole) for 10min. The number of Edu-positive cells was counted under a microscope in five random fields (×100). All assays were independently performed in triplicate.

### Colony formation assay

LN229 or U87 cells were plated in 6-well culture plates at 500 cells/well. Each cell group had two wells. After incubation for 10 days at 37°C, cells were washed twice with phosphate buffered saline and stained with hematoxylin solution. The number of colonies containing >50 cells was counted under a microscope. The colony formation efficiency was calculated as (number of colonies/number of cells inoculated) × 100%. All assays were independently performed in triplicate.

### Cell migration and invasion assays

For the cell migration assay, 1 × 10^4^ cells in 100μl medium without fetal bovine serum were seeded on a fibronectin-coated polycarbonate membrane insert in a transwell apparatus (Costar, Corning, NY, USA). In the lower chamber, 500μl medium with 10% fetal bovine serum was added as chemoattractant. After the cells were incubated for 6h at 37°C in a 5% CO_2_ atmosphere, the insert was washed with phosphate buffered saline, and cells on the top surface of the insert were removed with a cotton swab. Cells adhering to the lower surface were fixed with methanol, stained with crystal violet solution and counted under a microscope in five predetermined fields (×100). All assays were independently repeated at least thrice. The procedure for the cell invasion assay was similar to the cell migration assay, except that the transwell membranes were precoated with 24μg/μl matrigel (R&D Systems, Inc., Minneapolis, MN, USA) and the cells were incubated for 8h at 37°C in a 5% CO_2_ atmosphere. Cells adhering to the lower surface were counted the same way as the cell migration assay.

### Transient transfection

The miR-204-5p mimics, miR-204-5p inhibitor oligonucleotides and their corresponding negative control (miR-NC and anti-NC) were synthesized at Ribo Biotech (Guangzhou, China). Twelve hours prior to transfection, cells were plated onto a 6-well or a 96-well plate (Nest Biotech, Shanghai, China) at 30–50% confluence. Transfection of plasmids or oligonucleotides was performed using the Lipofectamine 2000 reagent (Invitrogen, Carlsbad, CA) according to the manufacturer’s instruction.

### 3′-UTR luciferase reporter assays

RAB22A, BCL2, CCPG1 and KLF12 were predicted to be possible target of miR-204-5p by RNAhybrid (The BiBiServ, Bielefeld, Germany) and miRwalk software (University of Heidelberg, Mannheim, Germany). RAB22A, BCL2, CCPG1 or KLF12 3′-UTR were cloned into psiCHECK-2 vectors (named wt). Site-directed mutagenesis of the miR-204-5p binding site in RAB22A, BCL2, CCPG1 or KLF12 3′-UTR were performed using GeneTailor Site-Directed Mutagenesis System (Invitrogen, Guangzhou, China; named mt). For reporter assays, wt or mt vector and the control vector psiCHECK-2 vector were cotransfected into LN229 cells with miR-204-5p mimics or inhibitors in 48-well plates, and then harvested for luciferase assay 48 h after transfection. Luciferase assays were performed by using the Dual-Luciferase Reporter Assay System (Promega Corporation, Madison, WI, USA) according to the manufacturer’s protocol. Firefly luciferase was used for normalization.

### Western blotting

The protein levels of RAB22A (Santa Cruz, CA) were determined by Western blotting in LN229 and U87 cells transfected with miR-204-5p mimic, miR-204-5p inhibitor, or the corresponding negative control (NC). β-Actin (Santa Cruz, CA) served as an internal control. Briefly, the protein concentration was determined using the BCA method. Equal quantities of proteins were separated by sodium dodecyl sulfate polyacrylamide gel electrophoresis (SDS-PAGE) and transferred by electroblotting onto a nitrocellulose membrane. Membranes were incubated with the primary antibody at 4°C overnight. After additional TBST washes, membranes were incubated with corresponding horseradish peroxidase-conjugated secondary antibodies (Bio-Rad) for 1 hr at room temperature and detected by the enhanced chemiluminescence method (SuperSignal West Pico substrate; Pierce; Rockford, IL).

### Tumor xenograft live imaging

All procedures and experiments involving animals in this study were performed in accordance with the National Institutes of Health Guide for Care and Use of Laboratory Animals. The study protocol was approved by the Animal Ethics Committee at Institute of Basic Medical Sciences, China. The IACUC committee members at Institute of Basic Medical Sciences approved this study. All efforts were made to minimize the number of animals used and their suffering. Female immunodeficient nude mice of 4–5 weeks of age were purchased from Shanghai SLAC Laboratory Animal Co., Ltd. (Shanghai, China) and were housed in the Animal Resource Facility. Tumor xenograft live imaging glioma xenografts stably expressing firefly luciferase together with miR-204 or the corresponding control vector were orthotopically implanted in the brains of nude mice. At day 5, 15 and 30, tumors were measured by fluorescent images of whole mice using an IVIS Lumina Imaging System (Xenogen).

### Immunohistochemistry

Immunohistochemistry assays were performed using antibodies against Ki67. After fixation, tumor sections (4 μm thick) were deparaffinized and rehydrated. Following rehydration, antigen retrieval was carried out by placing the slides in 10 mM sodium citrate buffer (pH 6.0) at 95°C for 20 min followed by 20-min cooling. The sections were then washed in PBS and non-specific binding sites were blocked with 1% bovine serum albumin with 2% goat serum in PBS before incubation with antibody. After washing, the sections were incubated with biotinylated secondary antibody followed by horseradish peroxidase-conjugated streptavidin. The sections were further incubated with 2,4-diaminobenzidine substrate and counterstained with hematoxylin. Each sample was examined separately and scored by two pathologists.

### Statistical analysis

Data were presented as mean ± SD unless otherwise indicated. The statistical significance of the difference between the values of control and treatment groups was determined by either Student *t* test or simple one-way ANOVA followed by Tukey's*post hoc* test for multiple comparisons using Prism version 5 (GraphPad Software, Inc.). The survival curves were plotted according to the Kaplan–Meier method, with the log-rank test applied for comparisons. Values of *p*<0.05 were considered statistically significant.

## Results

### Downregulation of miR-204-5p is correlated with the progression of primary gliomas

**Fig 1:**
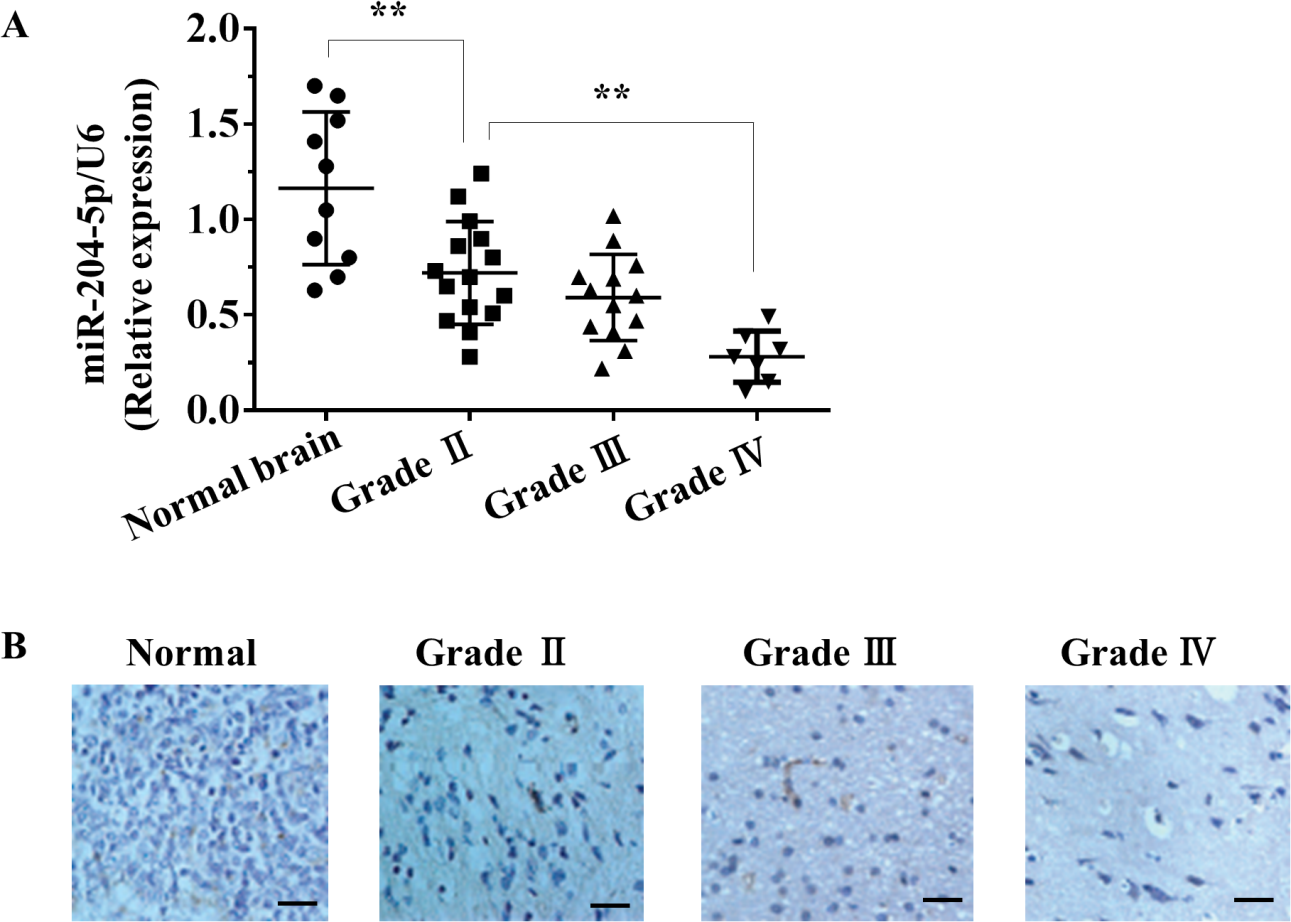
miR-204-5p is downregulated in glioma tissues. A, Decreased levels of miR-204-5p were positively correlated with the status of pathology classification by qRT–PCR). **, *p*< 0.01 compared with the control group. B, In situ hybridization assay showing the expression level of miR-204-5p in normal brain tissues and glioma tissues. Scale bars, 100 μm.

To identify the roles of miR-204-5p in the development of gliomas, we analyzed the expression level of miR-204-5p in 35 snap-frozen glioma tissues and 10 normal brain tissues by quantitative real-time PCR (qRT–PCR). Compared with normal brain tissues, significant downregulation of miR-204-5p was observed in glioma tissues (Fig 1A). We also observed that reduced levels of miR-204-5p in glioma patients were positively correlated with the status of pathology classification (WHO II vs WHO IV; p<0.01). Furthermore, *in situ* hybridization assay demonstrated miR-204-5p expression was significantly suppressed in glioma tissues compared with normal brain tissues. A gradually reduced miR-204-5p expression was found from grade II to grade IV samples (Fig 1B).

### Overexpression of miR-204-5p inhibits growth of glioma cells *in vitro*

**Fig 2:**
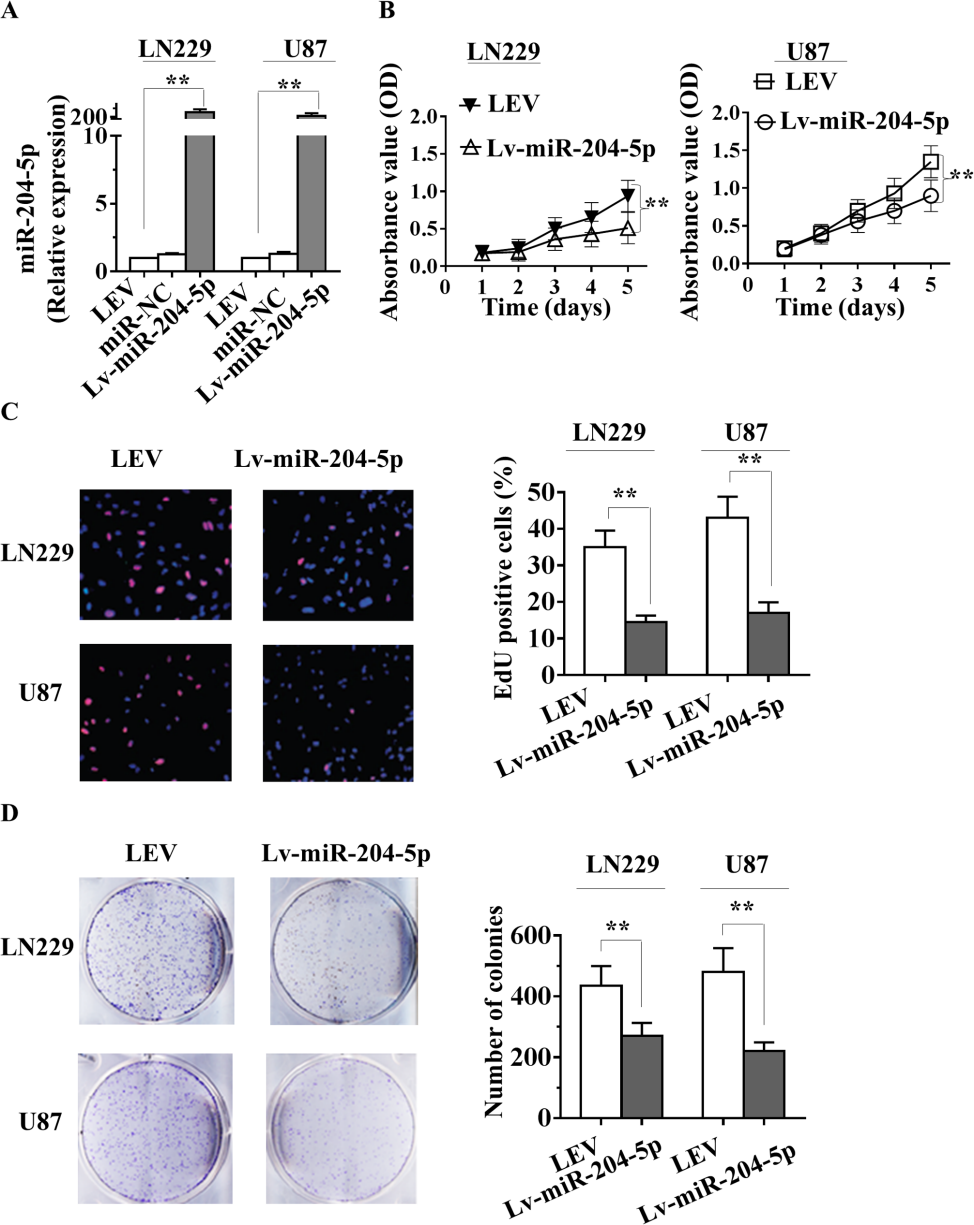
miR-204-5p inhibits glioma cell growth *in vitro*. A, More than 200-fold increase in the expression of miR-204-5p was observed in Lv-miR-204-5p-LN229 cells and Lv-miR-204-5p-U87 cells compared to the LEV group by qRT-PCR. B, Effect of Lv-miR-204-5p and controlled lentiviral empty vector (LEV) on LN229 and U87 cell viability was measured by MTT assay. Absorbance was read at 490nm with averages from triplicate wells. C, The effect of Lv-miR-204-5p or LEV on the proliferation of glioma LN229 and U87 cells was examined by EdU incorporation assay. D, *In vitro* proliferative ability of glioma cells was significantly decreased in overexpressed miR-204-5p cells compared with LEV cells by colony formation assay. Data are presented as mean ± SD from three independent experiments. **, *P*< 0.01 compared with the LEV control group.

To further explore its biological roles in glioma, miR-204-5p overexpression lentiviral vector (Lv-miR-204-5p), lentiviral empty vector (LEV) or negative control miRNA (miR-NC) were transfected into human glioma LN229 and U87 cells. As shown in Fig 2A, more than 200-fold increase in miR-204-5p expression was observed in Lv-miR-204-5p-LN229 cells and Lv-miR-204-5p-U87 cells compared with the LEV group by qRT–PCR. In addition, the miR-204-5p cotransfection at concentrations of 0, 1, 3 and 10.0 nM showed that the levels of miR-204-5p gradually increased with the increase of miR-204-5p mimics concentrations (S1 Fig). Subsequently, glioma cell growth was detected *in vitro*. Overexpression of miR-204-5p resulted in a significant decrease in viability compared with LEV group both in LN229 and U87 cells in an 3-(4, 5-dimethylthiazol-2-yl)-2, 5-diphenyltetrazolium bromide (MTT) assay (Fig 2B). Furthermore, EdU incorporation assays showed that the proliferation of Lv-miR-204-5p-LN229 and U87 cells were significantly inhibited by Lv-miR-204-5p relative to LEV (Fig 2C). Ectopic miR-204-5p expression blocked proliferation of glioma cells in colony formation assays (Fig 2D). These results demonstrated that miR-204-5p could exert a significant inhibitory effect on growth of glioma cells *in vitro*.

### MiR-204-5p expression inhibits migratory and invasive ability in glioma cells

**Fig 3:**
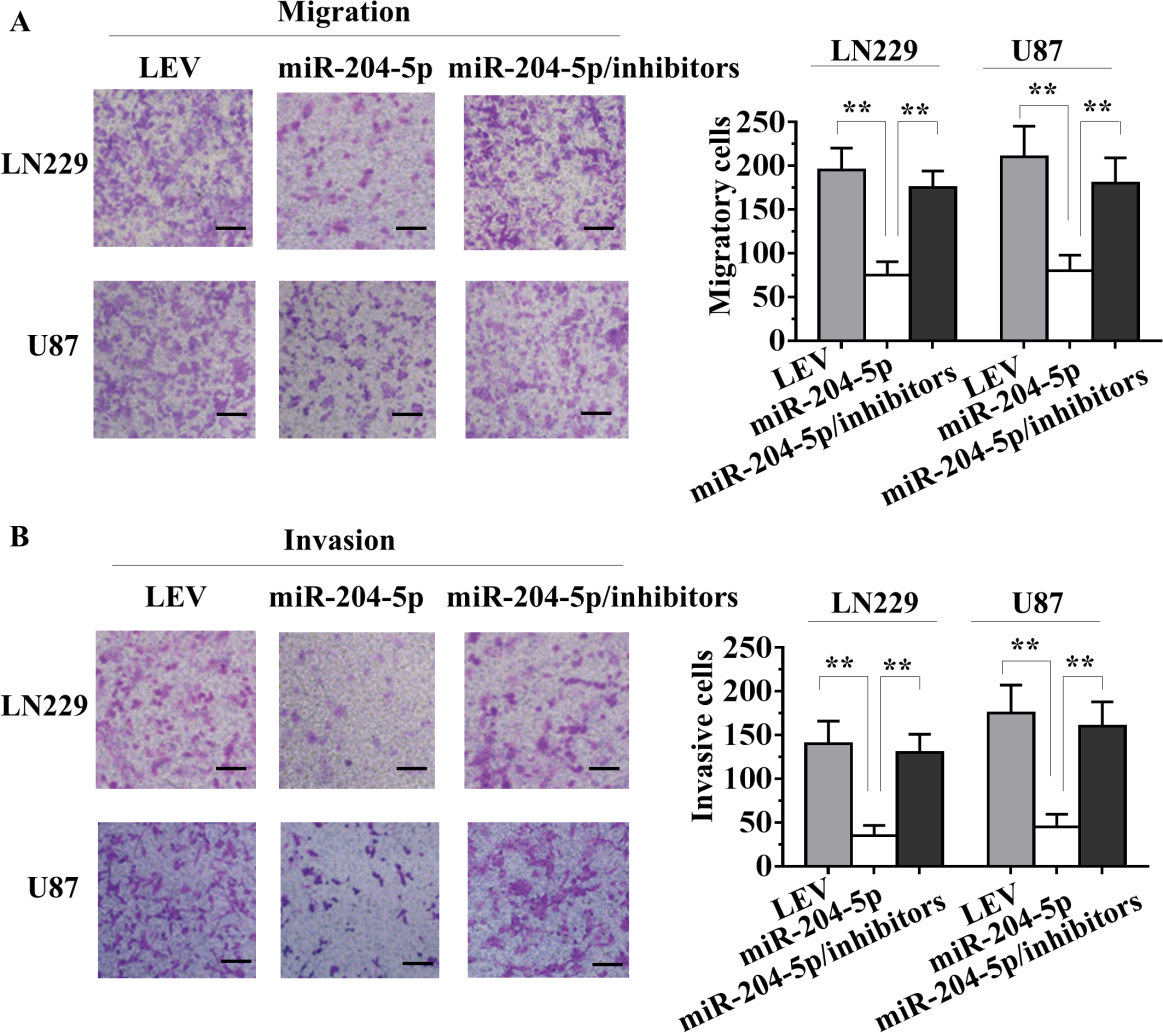
miR-204-5p inhibits migration and invasion of glioma cells *in vitro*. A, Stably upregulating miR-204-5p reduced the migration ability of LN229 and U87 cells *in vitro*, and knockdown of miR-204-5p in Lv-miR-204-5p cells rescued the migration ability. B, Stably upregulating miR-204-5p reduced *in vitro* invasiveness of LN229 and U87 cells, and specific inhibition of miR-204-5p in Lv-miR-204-5p cells rescued the invasion ability. Data are presented as mean ± SD from three independent experiments. **, *P*< 0.01 compared with the LEV control group. Scale bar, 100 μm.

To examine the effect of miR-204-5p on cell migration, using a transwell chamber, we determined changes in cell migration after 6h of incubation. Compared with the LEV cells, Lv-miR-204-5p-LN229 and Lv-miR-204-5p-U87 glioma cells both showed significantly decreased migratory ability (Fig 3A). In addition, to examine the effect of miR-204-5p on cell invasiveness, Lv-miR-204-5p and LEV cells were cultured in a Boyden chamber. After 8h of incubation, Lv-miR-204-5p-LN229 and Lv-miR-204-5p-U87 cells invaded through the matrigel, and a significant decrease was observed compared with control LEV cells (Fig 3B). Moreover, miR-204-5p inhibitors were transient transfected into LN229 and U87 cells. A significant difference in expression level of miR-204-5p was observed between the miR-204-5p inhibitors group and the negative control (NC) group by RT–qPCR. However, there was no significant difference in MTT assay results between miR-204-5p inhibitor group and NC control group (S1 Fig). Furthermore, Lv-miR-204-5p/inhibitor cells, LEV cells, miR-204-5p inhibitor cells and NC cells were cultured in transwell and Boyden chamber, respectively. No significant difference was observed between miR-204-5p inhibitor and NC groups (S1 Fig). However, a significant increase of cell invasion and migration was found in Lv-miR-204-5p/inhibitor group compared with the Lv-miR-204-5p group (Fig 3A and 3B). Together, these data suggest that miR-204-5p inhibits glioma cells growth by inhibiting cell metastasis.

### MiR-204-5p directly targets to the RAB22A 3′UTR

**Fig 4:**
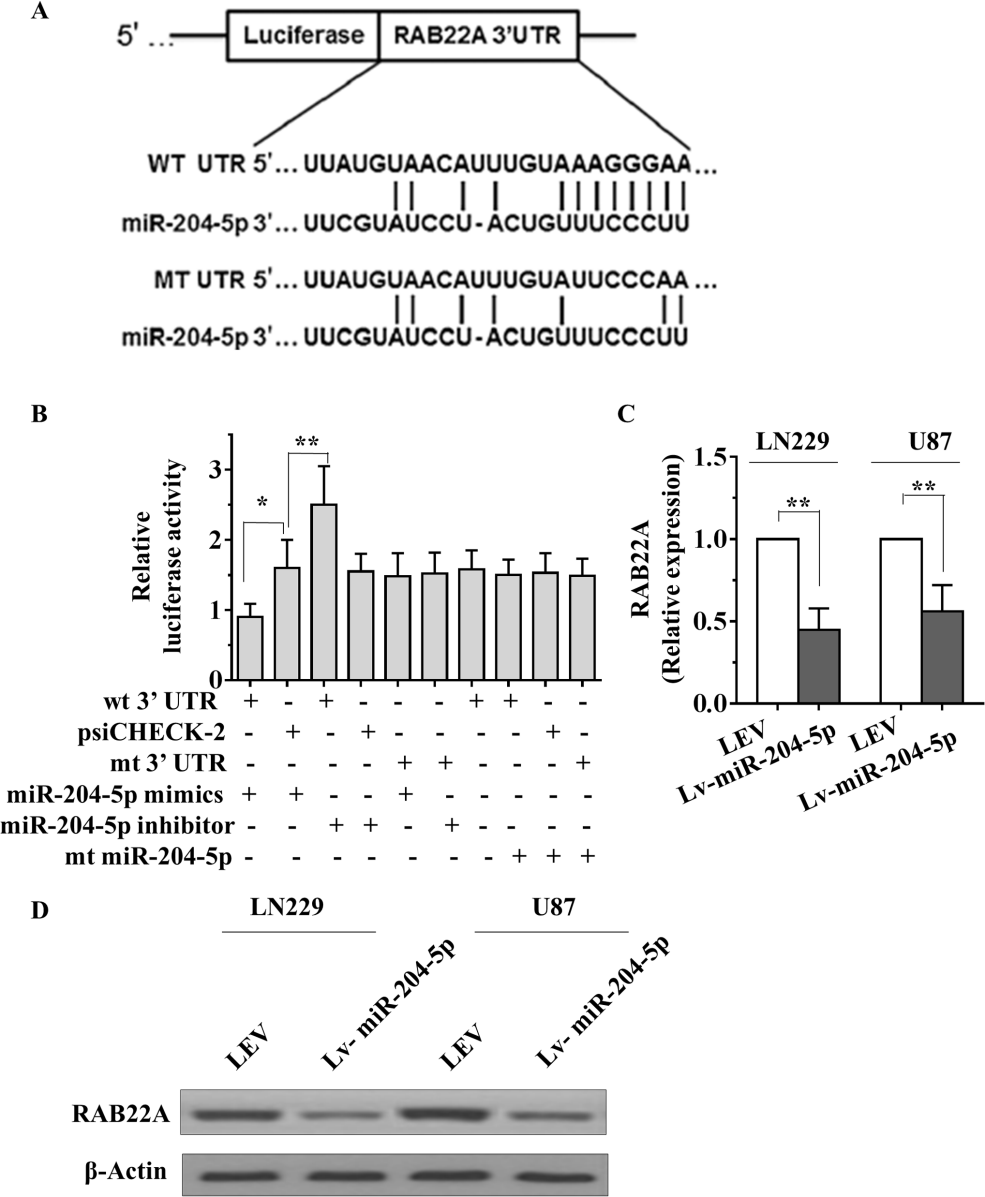
miR-204-5p directly inhibits expression of RAB22A via its 3′-UTR. A, Sequence alignment between miR-204-5p and the 3′-UTR of human RAB22A mRNA. B, The effect of miR-204-5p on the activity of firefly luciferase reporter containing either wild type or mutant type 3′-UTR was tested using luciferase reporter gene assays. C and D, The effect of miR-204-5p on the endogenous expression levels of RAB22A was examined in LN229 and U87 cells by qRT–PCR (C) and Western blot (D) analysis. Data are presented as mean ± SD from three independent experiments. **, *P*< 0.01 compared with the control group.

To delineate the mechanism by which miR-204-5p inhibited cell proliferation, invasion and migration in glioma cells, miR-204-5p target genes were searched using the TargetScan (http://www.targetscan.org/) and PicTar (http://pictar.mdc-berlin.de/) algorithms. Based on the presence of miR-204-5p sites in their 3′-untranslated region (3′-UTR), >100 messenger RNAs (mRNAs) were predicted to be regulated by miR-204-5p. Because it is generally accepted that miRNAs exert their function by inhibiting the expression of their target genes, miR-204-5p may execute its tumor-suppressive function by downregulating targets that normally have tumor-promoting functions. On the basis of this rationale, 4 candidate genes (RAB22A, BCL2, CCPG1 and KLF12) were selected from the downregulated genes. Among these candidates, RAB22A is a member of the RAB family that plays a role in the endocytic pathway [32]. Previous studies have reported that RAB22A is an essential component involved in the promotion of cell proliferation, migration and invasion in several types of tumors [33, 34]. Luciferase reporter assays were then conducted to determine the influence of miR-204-5p on the expression of these 4 genes. The results revealed that miR-204-5p could inhibit the expression of the reporter gene in recombinant plasmids containing the 3′UTRs of RAB22A, BCL2 and CCPG1, particularly RAB22A (S2 Fig). To further investigate whether miR-204-5p mediated the expression of RAB22A in glioma cells. We subcloned 3′-UTR region of RAB22A mRNA including the predicted miR-204-5p recognition site (wild type) or the mutated sequence (mutant type) into luciferase reporter plasmids. Our results showed that the reporter plasmid with 3′-UTR of RAB22A resulted in a significant decrease in luciferase activity after transfection with miR-204-5p mimic, whereas the plasmid without RAB22A 3′-UTR had no change in luciferase activity (Fig 4A and 4B). Subsequently, mRNA and protein levels of RAB22A in Lv-miR-204-5p-treated cells were detected using qRT–PCR and Western blot. Overexpression of miR-204-5p significantly decreased both the RAB22A mRNA and protein levels compared with LEV cells (Fig 4C and 4D).

### Role of miR-204-5p in tumorigenesis via directly targeting RAB22A

**Fig 5:**
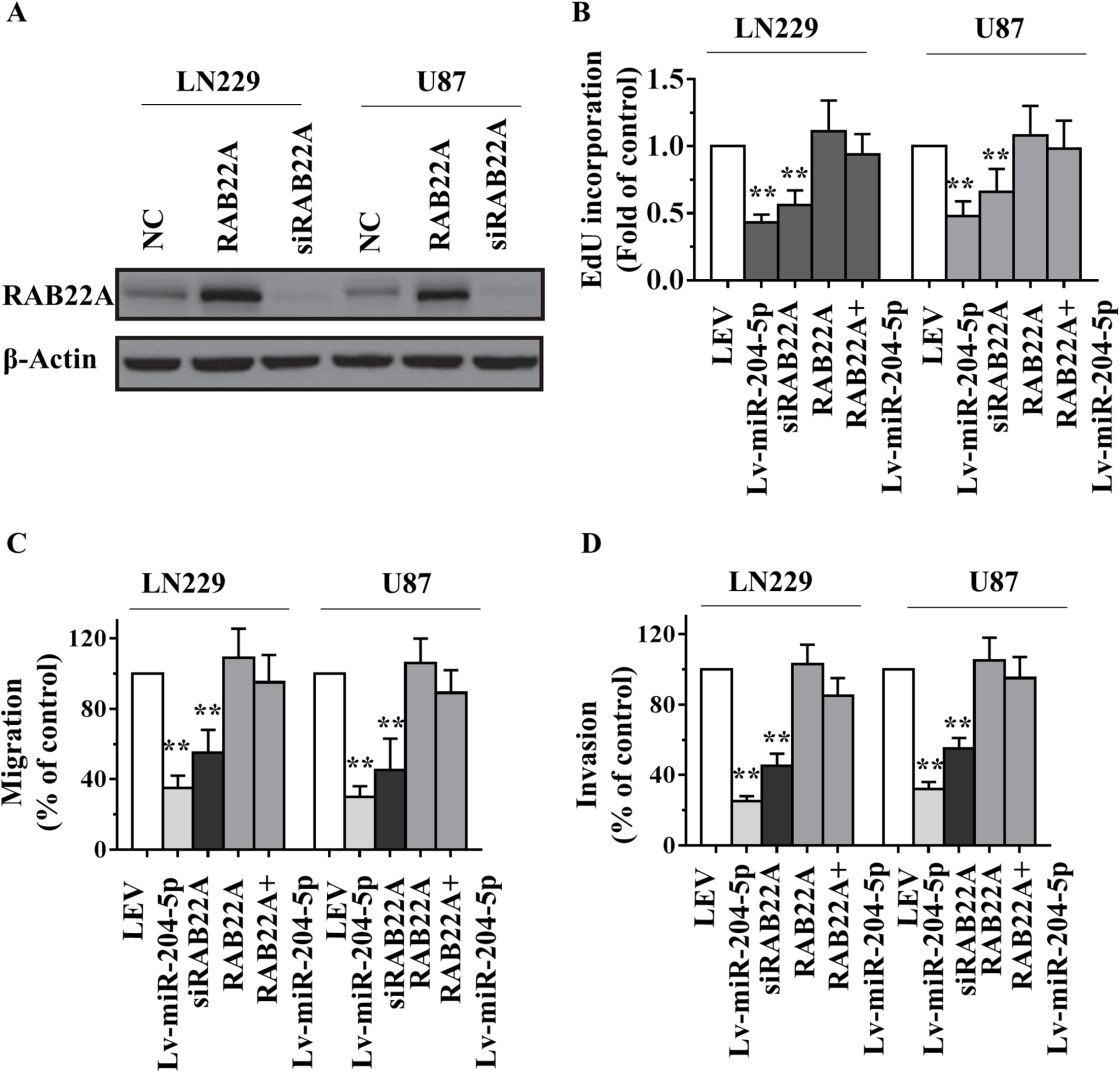
miR-204-5p represses glioma cells proliferative, migration and invasion by targeting RAB22A. A, The expression level of RAB22A was examined by Western blot analyses in LN229 or U87 cells treated with ectopic RAB22A or siRNAs targeting RAB22A. B-D, EdU incorporation assays (B), transwell migration assays (C) and Boyden invasion assays (D) of LN229 or U87 glioma cells were performed after transfection with NC, siRNA against RAB22A and/or miR-204-5p as indicated. Data are presented as mean ± SD from three independent experiments. **, *P*< 0.01 compared with the control group.

To determine the functional significance of RAB22A in the miR-204-5p–induced phenotype, we performed a series of restoration assays using LN229 and U87 cells. We utilized an expression construct that encodes the entire RAB22A coding sequence but lacks the 3′-UTR. We then inserted this sequence into a plasmid (plasmid-RAB22A), and simultaneously suppressed RAB22A with specific small-interfering RNA (siRNA)—siRAB22A. The expression of RAB22A protein in plasmid-RAB22A-treated cells was upregulated by 200–300% compared with cells transfected by plasmid empty vector. Western blot analysis also demonstrated that siRAB22A was able to effectively knockdown the expression of RAB22A in glioma LN229 and U87 cells (Fig 5A). Further functional studies demonstrated that knockdown of RAB22A produced similar changes in the proliferative, migratory and invasive capacity assay compared with that of Lv-miR-204-5p groups. Likewise, RAB22A overexpression could restore miR-204-5p-mediated reduction of growth, migration and invasion (Fig 5B–5D). To further determine the correlation between miR-204-5p and RAB22A expression, we analyzed expression levels of miR-204-5p and RAB22A in human GBM tissue specimens by qRT-PCR. As shown in S1 Table, low expression levels of miR-204-5p glioma contained comparatively high RAB22A expression than those in high miR-204-5p expression specimens. Taken together, these results imply that miR-204-5p exerts tumor-suppressive function in glioma cells via directly targeting RAB22A.

### MiR-204-5p suppresses tumorigenicity of glioma cells *in vivo*

**Fig 6:**
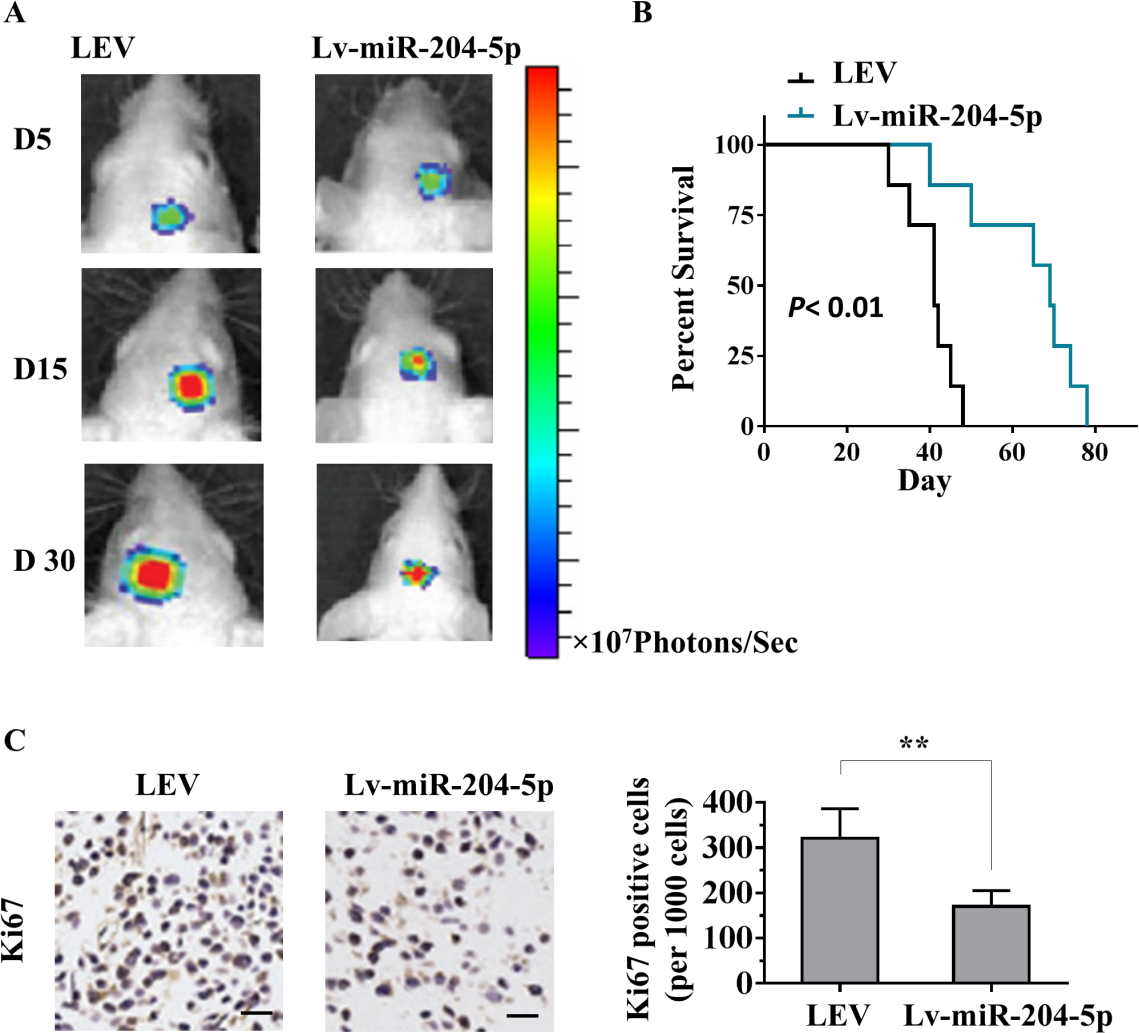
Reintroduction of miR-204-5p suppresses glioma tumorigenesis *in vivo*. A, Mice with intracranial glioma xenografts were monitored by luciferase live imaging system at indicated time points. Heat map scale bar represents photon emission. B, Survival curves of mice (seven mice per group) with brain glioma xenograft formed by Lv-miR-204-5p-LN229 or LEV-LN229 cells. C, Representative photomicrographs of tumor sections following IHC analysis for Ki-67 expression. Scale bars, 100 μm.

Having established that miR-204-5p inhibits glioma cells proliferation and invasion *in vitro*, we evaluated the growth inhibitory effect of miR-204-5p *in vivo* by using experimental intracranial models. We chose LN229 cell for the *in vivo* models. When the mice intracranially transplanted with Lv-miR-204-5p-LN229 that stably express luciferase and miR-204-5p, or control lentiviral empty vector (LEV), bioluminescence imaging was done for the whole body. As shown in Fig 6A, Lv-miR-204-5p-LN229 cell lines exhibited significantly slower growth after implantation. For the highly proliferative LN229 cells, results from luminescence images revealed a high degree of suppression on the growth of Lv-miR-204-5p-LN229 cells in contrast to the larger intracranial tumor formed by the control cells at day 15 after implantation. To analyze the survival times of the treatment groups, we generated Kaplan-Meier survival curves (Fig 6B), which demonstrated that miR-204-5p significantly prolonged survival compared with control group. Furthermore, Ki-67 staining showed that tumors of Lv-miR-204-5p cells had fewer proliferative cells than LEV transfected tumor cells (Fig 6C). These results demonstrated that miR-204-5p could exert a significant inhibitory effect on tumorigenesis of glioma cells *in vivo*.

## Discussion

Glioma is the most common primary brain tumor and is produced by brain and spinal cord glial cells. Similar to other tumors, glioma mainly results from genetic risk factors and environmental carcinogenic factors. Some genetic diseases, such as neurofibromatosis and tuberous sclerosis disease, are predisposing factors for glioma, and the chances of glioma are much higher in patients with these diseases than in the general population [35]. In addition, some environmental carcinogenic factors may also be associated with the occurrence of glioma [36]. miRNAs have emerged as key factors involved in several biological processes, including development, differentiation, cell proliferation, and tumorigenesis [37, 38]. The dysregulation of miRNAs in cancer has been repeatedly described, for example, in prostate, bladder, kidney cancer [39], breast cancer [40], and colon cancer [41]. Thus, it is possible that microRNA (miRNA), a highly conserved gene regulatory factor, may shed light on the potential mechanism of glioma. In this study, we found that expression of miR-204-5p was decreased in glioma tissues compared with normal brain tissues. We further found that miR-204-5p expression was negatively correlated with tumor grading. These results suggest that miR-204-5p may function as a suppressor in gliomas.

Alternations in miRNA expression have been implicated in the initiation, progression, and metastasis of a number of human cancers [42]. MiRNAs consist of a class of small, non-coding RNAs (19–25 nucleotides) that function as post-transcriptional gene regulators [6, 43, 44]. They are primarily negative gene regulators of post-translational repression. Although the biological functions of most microRNAs have not been completely revealed, several studies have demonstrated that aberrant expression levels of microRNAs are involved in glioblastoma initiation and progression. It has been shown that microRNAs may function as oncogenes or tumor suppressor genes by regulating the expressions of the functional genes [45]. For example, miR-21 is highly overexpressed in glioblastoma and has important roles in cellular proliferation, invasion, and apoptosis by regulating the EGFR signaling pathway [46, 47]. Important down regulated microRNAs have also been identified in glioblastoma, such as miR-124, miR-128 and miR-137 [48–50]. miRNAs may negatively control downstream targeted genes to interfere with cell activities. Therefore, the expressions and functional roles of miRNAs are very important in tumor development. miRNAs have been shown to negatively regulate the post-transcriptional expression of their target genes, which include tumor suppressors and oncogenes [45]. miR-21 was found to be highly expressed in colorectal cancers, and induced metastasis and invasion by inhibiting the tumor suppressing gene PDCD4 in colorectal cancer cells [51]. Thus, as an oncogene, miR-21 exhibited regulatory effects in many instances of cancer development. However, when expressed at lower levels in tumor, miRNAs also demonstrated tumor suppressing functions. For example, the action of let-7 miRNA as a tumor suppressor was found to be absent in lung cancers and to promote cancer progression by inhibiting downstream translation of MYC [52]. In the current study, we focused on miR-204-5p. miR-204-5p has been reported to function as a tumor suppressor in a variety of human cancers through different mechanisms, suggesting its extensive function in tumorigenesis. For example, miR-204 inhibited tumor growth in renal clear cell carcinoma [53], suppressed invasion in endometrial cancer [54], gastric cancer [55], and head and neck tumor [56]. In addition, miR-204 could affect chemoresistance in neuroblastoma and gastric cancer cells by targeting BCL2 [54, 57]. However, the role of miR-204-5p in glioma was not well known. In this study, our data affirmed that ectopic expression of miR-204-5p inhibited the growth of glioma cells, suggesting that the downregulation of miR-204-5p may be responsible for the enhanced growth of glioma cells.

In the subsequent mechanistic study, we demonstrated that miR-204-5p directly targets RAB22A to inhibit proliferation, migration and invasion in glioma cells. In miR-204-5p–transfected cells, ectopic RAB22A expression can rescue the proliferation, migration and invasion ability attenuated by miR-204-5p. Li and colleagues performed preliminary luciferase reporter assay to validate the targeting of miR-204-5p to the 3′UTRs of RAB22A under stress conditions in human trabecular meshwork cells [58]. RAB proteins, including more than 60 members in mammals, constitute a Ras superfamily of GTPases [59]. They are usually activated by binding GTP in the transport vesicles and then hydrolysed to generate GDP-bound RABs after membrane fusion [60]. Recent data suggest an emerging role for RAB GTPases in human cancer [61]. RAB22A is a less studied member of the RAB family that plays a role in the endocytic pathway [32]. Although RAB22A has recently been reported to be upregulated in hepatocellular carcinoma, cholangiohepatoma [62], melanoma [63] and colorectal cancer [29], little is known about its role in human tumorigenesis, especially in human glioma. Here, we revealed that RAB22A overexpression could significantly increase cell growth, migration and invasion. These data suggest that RAB22A is a potential new oncogene and prognostic factor for glioma. The detailed role and mechanism by which RAB22A promotes glioma development and progression should be investigated in future work. The extremely poor prognosis of patients with gliomas is largely due to the high tendency of tumor invasiveness, unlimited proliferation, and abnormal regulation of cell apoptosis that lead to severe structural and functional damage to the surrounding brain tissue and occurrence and progression of tumor [64]. As a result of our present study, we have made clear that reintroduction of miR-204-5p dramatically repressed the migration and invasion of glioma cells. *In vivo*, miR-204-5p transfected LN229 cells displayed a marked reduction of the tumor. Kaplan-Meier survival analysis demonstrated that miR-204-5p significantly prolonged survival of glioma in mice. Taken together, miR-204-5p may play an important functional role in decreasing the ability of neoplastic cells to grow and invade tissue in glioma.

In summary, our study demonstrated that the expression of miR-204-5p is significantly downregulated in clinical glioma tissues. Overexpression of miR-204-5p is able to suppress glioma cell growth and metastasis through directly targeting RAB22A. Therefore, miR-204-5p may be considered to be a tumor suppressor and has significant value as an unfavorable progression indicator for glioma patients, and may serve as a therapeutic target in the future.

## Supporting Information

S1 Fig Dosage effect of miR-204-5p overexpression and the function of knockdown miR-204-5p in glioma LN229 and U87 cells. A, LN229 and U87 cells were transfected with different concentrations of miR-204-5p mimics (0, 1, 3 and 10.0 nM), the levels of miR-204-5p were measured by qRT-PCR. B, LN229 and U87 cells were transfected with miR-204-5p inhibitors or negative control miRNA (NC), the levels of miR-204-5p were detected by qRT-PCR. C, Effect of miR-204-5p inhibitors on LN229 and U87 cell growth was measured by MTT assay. Absorbance was read at 490 nm with averages from triplicate wells. D, Effect of specific knockdown of miR-204-5p on LN229 and U87 migration and invasion was detected by Transwell and Boyden chamber assay. Data are presented as mean ± SD. **, *P*< 0.01 compared with the control group. Scale bars, 100 μm.
(TIF)

S2 Fig Screening for candidate target genes of miR-204-5p in glioma. Four downregulated genes (RAB22A, BCL2, CCPG1 and KLF12) were selected from the downregulated genes in the initial screening based on the functional analysis of these genes, and their 3′UTRs were assessed using the luciferase reporter assay. Data are presented as mean ± SD. **, *P*< 0.01 compared with the control group.
(TIF)

S1 Table The correlation between miR-204-5p and RAB22A expression in glioma samples. (PDF)
