# Supplementary figures and images for: Decreased Expression of MiRNA-204-5p Contributes to Glioma Progression and Promotes Glioma Cell Growth, Migration and Invasion
Source: PLoS One. 2015 Jul 2;10(7):e0132399. doi: 10.1371/journal.pone.0132399 (PMC4489611; doi:10.1371/journal.pone.0132399)

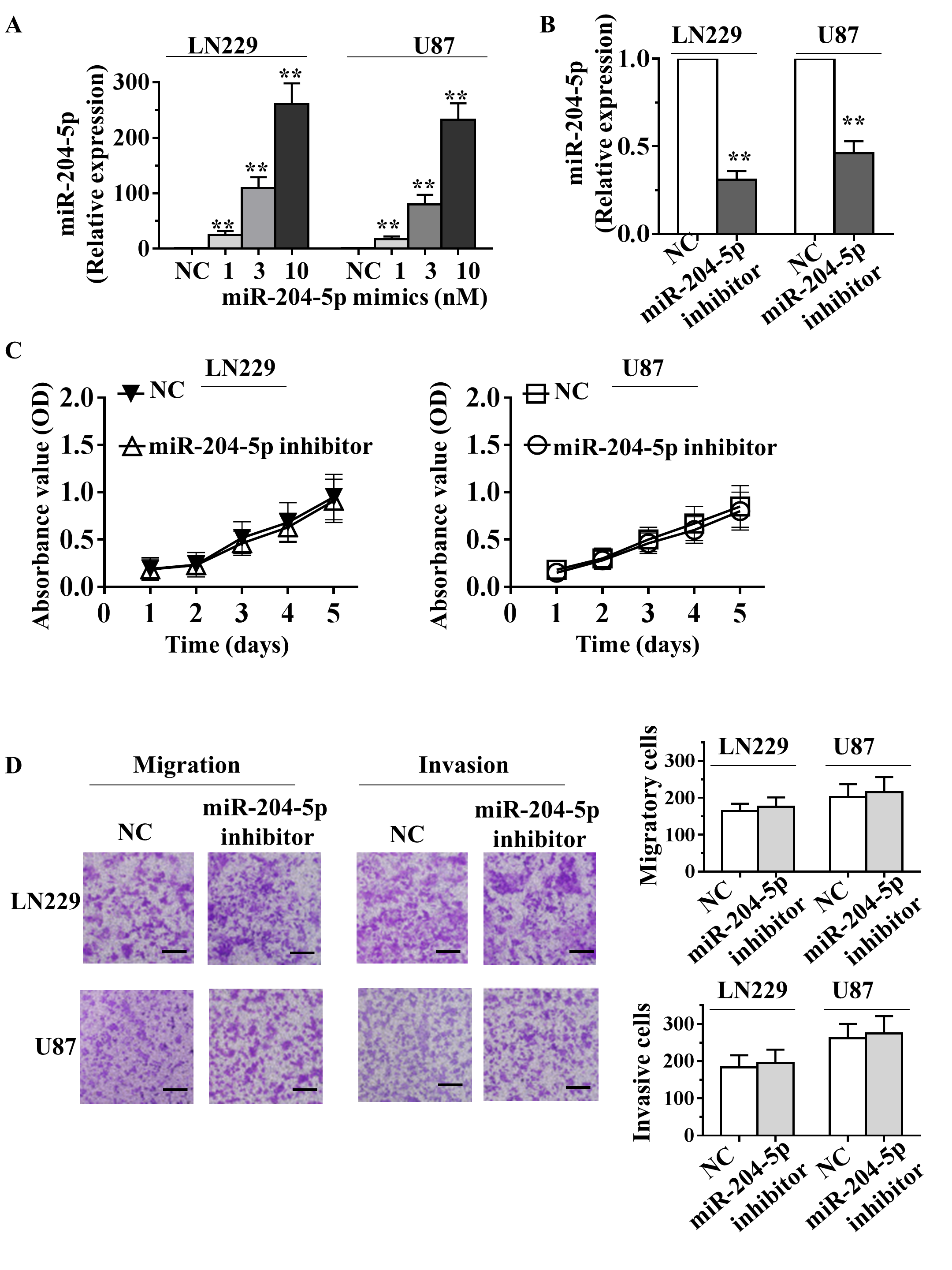

Supplement: S1 Fig — A, LN229 and U87 cells were transfected with different concentrations of miR-204-5p mimics (0, 1, 3 and 10.0 nM), the levels of miR-204-5p were measured by qRT-PCR. B, LN229 and U87 cells were transfected with miR-204-5p inhibitors or negative control miRNA (NC), the levels of miR-204-5p were detected by qRT-PCR. C, Effect of miR-204-5p inhibitors on LN229 and U87 cell growth was measured by MTT assay. Absorbance was read at 490 nm with averages from triplicate wells. D, Effect of specific knockdown of miR-204-5p on LN229 and U87 migration and invasion was detected by Transwell and Boyden chamber assay. Data are presented as mean ± SD. **, P< 0.01 compared with the control group. Scale bars, 100 μm. (TIF) [file pone.0132399.s001.tif]

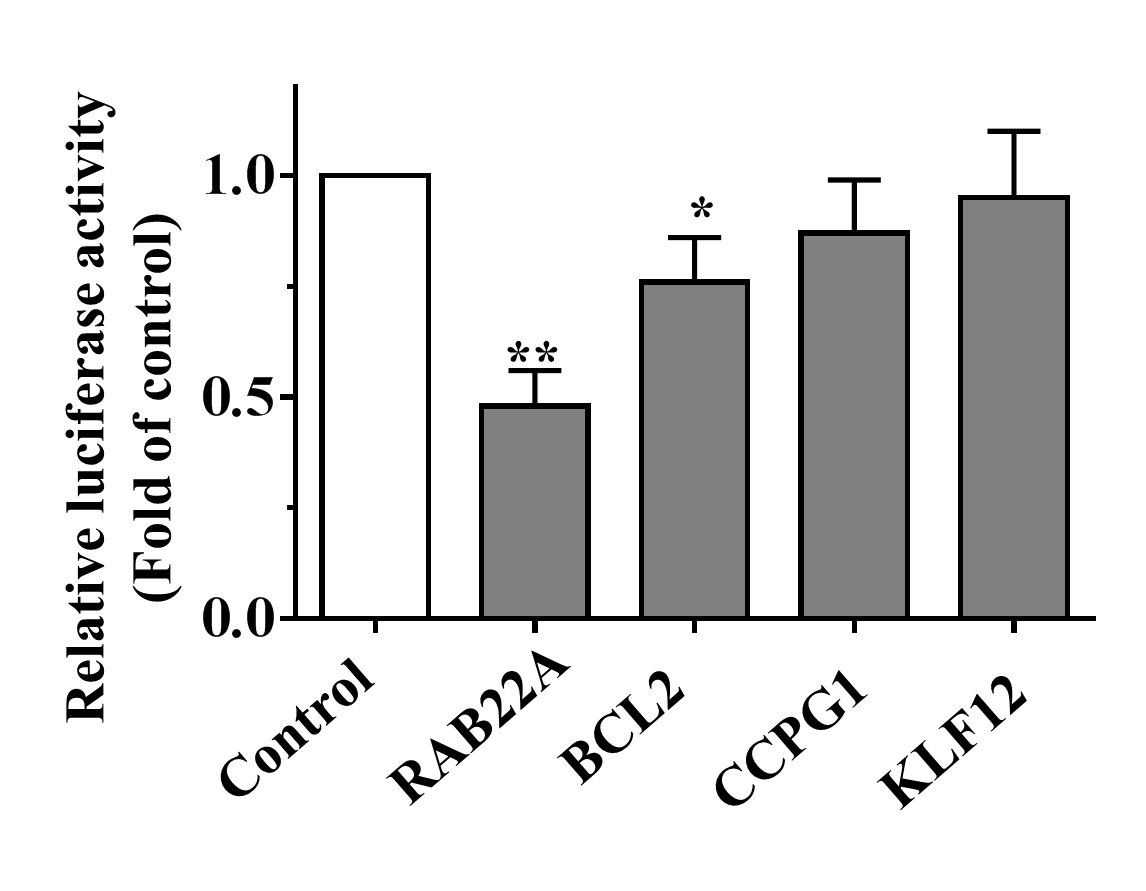

Supplement: S2 Fig — Four downregulated genes (RAB22A, BCL2, CCPG1 and KLF12) were selected from the downregulated genes in the initial screening based on the functional analysis of these genes, and their 3′UTRs were assessed using the luciferase reporter assay. Data are presented as mean ± SD. **, P< 0.01 compared with the control group. (TIF) [file pone.0132399.s002.tif]
