# Supplementary material for: Decreased Expression of MiRNA-204-5p Contributes to Glioma Progression and Promotes Glioma Cell Growth, Migration and Invasion
Source: PLoS One. 2015 Jul 2;10(7):e0132399. doi: 10.1371/journal.pone.0132399 (PMC4489611; doi:10.1371/journal.pone.0132399)
Supplement: S1 Table — (PDF) [file pone.0132399.s003.pdf]

**S1_ Table**. The correlation between miR-204-5p and RAB22A expression in glioma samples

| | | RAB22A | | Total |
|---|---|---|---|---|
| | | High | Low | |
| miR-204-5p | High | 2 (25.0%) | 6 (75.0%) | 8 |
| | Low | 22 (81.5%) | 5 (18.5%) | 27 |
| Total | | 24 | 11 | 35 |

The expression levels of miR-204-5p and RAB22A in human GBM tissue specimens were measured by qRT-PCR.
